# Clinical and Radiographic Success of Selective Caries Removal to Firm Dentin in Primary Teeth: 18-Month Follow-Up

**DOI:** 10.1155/2018/9213681

**Published:** 2018-03-26

**Authors:** Tássia Carina Stafuzza, Luciana Lourenço Ribeiro Vitor, Daniela Rios, Thiago Cruvinel Silva, Maria Aparecida Andrade Moreira Machado, Thais Marchini Oliveira

**Affiliations:** ^1^Department of Pediatric Dentistry, Orthodontics, Public Health, Bauru School of Dentistry, University of São Paulo, Bauru, SP, Brazil; ^2^Hospital for the Rehabilitation of Craniofacial Anomalies, University of São Paulo, Bauru, SP, Brazil

## Abstract

The selective caries removal is increasingly spreading in daily clinical practice because this minimally invasive technique treats deep carious lesion and decreases the risk of pulp exposure. This case report was aimed at describing the selective removal to firm dentin on the primary mandibular left first molar of a girl aged 7 years and 6 months. The Mineral Trioxide Aggregate (MTA Angelus™) was used as liner, and the tooth was definitively restored with resin-modified glass ionomer cement (Vitremer™). The clinical and radiographic following-up was performed at 6, 12, and 18 months after treatment. The treatment showed satisfactory results after 18-month following-up, suggesting that this minimally invasive approach for carious lesion removal can replace the total removal, when properly indicated. Notwithstanding, further randomized clinical trials with longer following-up periods are still necessary.

## 1. Introduction

Currently the literature reports many studies on selective caries removal [1–6]. The selective removal to firm dentin [7] enables the change in the carious lesion microenvironment, decreases the number and bacterial diversity which stops the carious lesion progression, reduces the risk of pulp exposure [8], and preserves pulp vitality [6]. Although growing evidences indicate that minimally invasive approaches are effective to treat carious lesions [9], selective caries removal success may depend on the appropriate use of liners and restorative materials.

Many bacteriostatic, bactericidal, and remineralizing materials can be applied to the remaining dentin after selective caries removal [1, 10], but no consensus exist on which liner would be the most suitable for teeth undergoing selective caries removal [5, 10]. Randomized clinical trials and systematic reviews are necessary to clarify the persistent doubts.

Faced with this reality, caries tissue management techniques have been widely discussed [5, 9, 11]. Thus, this case report describes the selective removal to firm dentin in the primary mandibular left first molar of a girl aged 7 years and 6 months using the Mineral Trioxide Aggregate (MTA Angelus) as a pulp protective material.

## 2. Case Report

**Figure 1:**
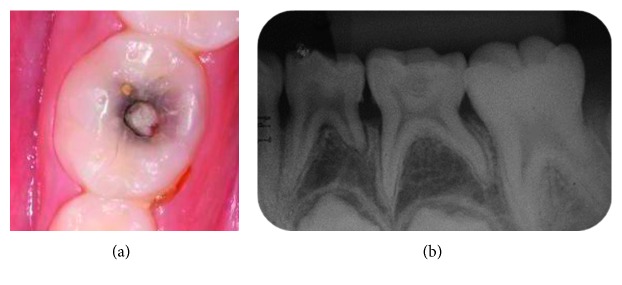
(a) Initial clinical aspect of the primary mandibular left first molar. (b) Initial periapical radiograph of the primary mandibular left first molar. Note the proximity of the carious lesion to the pulp.

A girl aged 7 years and 6 months searched treatment at the Pediatric Dentistry Clinic of the Bauru School of Dentistry, University of São Paulo. During the anamnesis, the mother reported no spontaneous pain in the primary mandibular left first molar what was confirmed by the girl during the clinical examination. In the clinical and radiographic examination, the presence of deep occlusal carious lesion close to the pulp was observed (Figures 1(a) and 1(b)).

First, topical anesthesia was performed with benzocaine (Benzotop DFL) applied to the previously dried mucosa with the aid of a cotton pellet. Local infiltrative anesthesia of inferior alveolar nerve was performed with articaine 4% and epinephrine 1 : 100.000 [12–15]. After rubber dam isolation, the total caries removal was performed on the lateral walls of the cavity with low-speed round burs (sizes 4, 5, and 6, KG Sorensen, São Paulo, Brazil). Selective caries removal was executed on pulpal wall with hand excavators until firm dentin, and it was possible to observe that the remaining dentin had a darker color and leathery consistency. Before applying the protective material, the cavity was cleaned and dried with sterile cotton pellet. The MTA (Angelus, Londrina, PR, Brazil) was mixed according to the manufacturer's instructions on a sterile glass plate and directly placed on the pulpal wall reaching a thickness of 2 mm. The resin-modified glass ionomer cement (RMGIC) (Vitremer, 3M/ESPE, Minnesota, USA) was mixed at 1 : 1 powder/liquid ratio and applied to the cavity with a Centrix syringe (Centrix™, Nova DFL, Taquara, Rio de Janeiro, RJ, Brazil). At the end, the patient's occlusion was checked.

**Figure 2:**
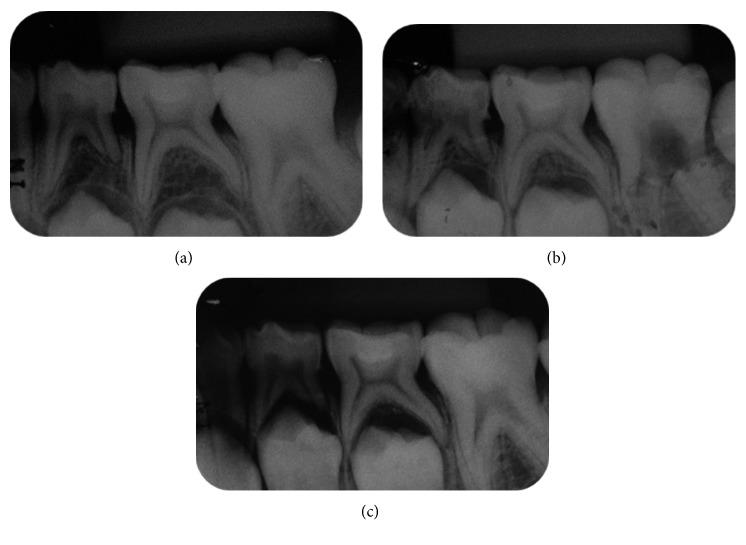
Radiographic following-up: (a) 6 months, (b) 12 months, and (c) 18 months.

**Figure 3:**
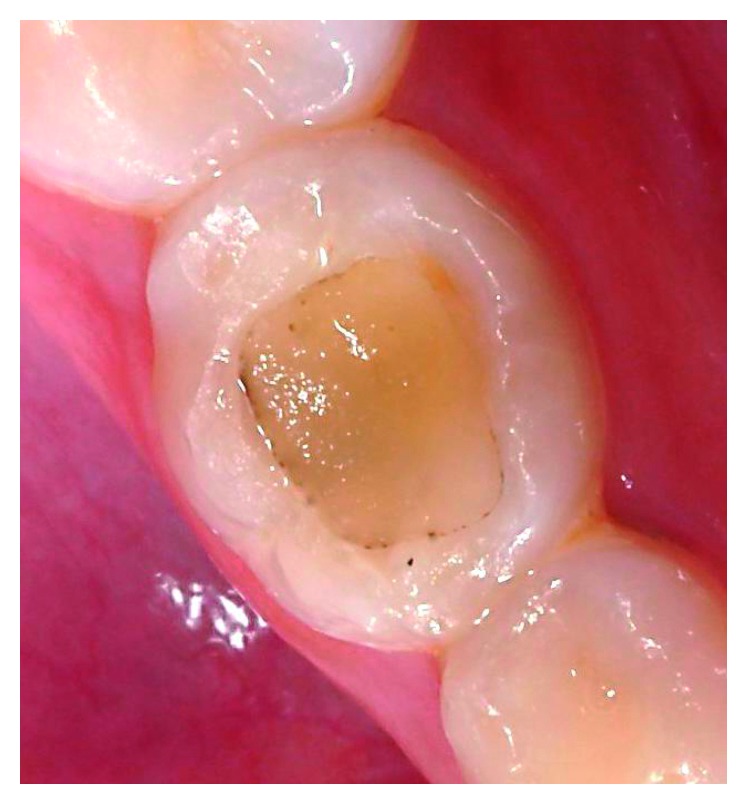
Clinical aspect at 18 months of follow-up.

During the 18-month following-up period, no pain, mobility, sensitivity to percussion, abscess/fistula, restoration failure (such as partial fracture or loss of restoration), internal/external resorption, signs of furcal impairment, and advanced rhizolysis stage were observed (Figures 2(a)–Figures 2(c) and 3).

## 3. Discussion

With the diagnosis of deep caries lesion without pulp involvement, there were two possible treatment options: total or selective caries removal. Total caries removal could lead to pulp exposure. In addition, the removal of all carious dentin from the cavity is no longer necessary to manage the carious lesion [9]. So, for this case, we choose the selective caries removal to avoid greater damage to the tooth [1–3, 9] and to minimize the potential complications of complete excavation of carious dentin close to the pulp by avoiding pulpotomy and a breach in the pulp [1–3].

Another advantage of the proposed treatment is the permanence of the primary teeth in the oral cavity until its natural exfoliation period [16], a fact that should be highlighted in the dental treatment of children. In this case report, the patient was seven and a half years old, which suggests that the primary molar will remain for at least 3-4 years in her mouth. With all this preexfoliation time, it has become imperative to perform a minimally invasive treatment that preserves the prognosis of dental longevity.

Scientific evidence reported significant chemical and morphological differences between dentin in permanent and primary teeth. The diameter of the dentinal tubules is larger in the primary teeth. This structural feature suggests that primary teeth may be more susceptible to sensitivity and transmission of harmful substances to pulp [17]. Thus, to ensure a correct indication of the therapy, the first primary molar treated in this case report did not present painful symptomatology, periapical lesion, fistula, or abscess, keys to success in minimally invasive dentistry.

The carious dentin is divided into two parts: the outermost part, infected dentin, which must be removed, and the innermost part, affected dentin, which should be preserved because it can be remineralized [18]. The main difficulty of selective removal to firm dentin is the subjective criteria to evaluate the amount of carious tissue to be removed [3]. To overcome this problem, the literature has determined that the best guide to be given is the operator's tactile feel [7]. In this case report, the dentin was removed until a harder tissue with leather consistency was found based on tactile perception of the operator [3, 7]. No caries detector dye was used.

The ideal liner to be placed on the remaining dentin after selective caries removal is still a subject that raises many doubts. Good results were obtained in studies that used calcium hydroxide as liner material [19–22], but the histological evaluation demonstrated that a lower inflammation and hyperemia of pulp and a thicker dentin bridge with formation of odontoblastic layer are more frequent with the use of MTA [23]. It can maintain the vitality of the remaining pulp, allowing it to continue to exhibit normal physiological functions [24, 25]. Thus, current clinical trials should evaluate the combined effect of carious tissue removal strategies with restorative procedures, including the use of liners [5]. MTA is a biocompatible material with satisfactory physical and chemical properties [24], and it has been constantly used in indirect pulp treatments of permanent and primary teeth [1, 26, 27, 28].

Currently, the literature lacks scientific evidence on the suitable restorative material for selective removal to firm dentin. Thus, ionomeric cements were chosen because they are less sensitive to the technique and can be placed in the cavity in only one increment [29]. Furthermore, RMGIC enables repair in case of failures in the restorations, assuring the minimally invasive approach [29].

In this case report, we applied a methodology standardized by the literature to evaluate the dentin-pulp complex response [1, 4]. Clinical and radiographic follow-up was performed at 6, 12, and 18 months [15]; however, other studies have follow-up times with longer evaluation periods [2, 3, 6]. In this way, the follow-up of this clinical case will continue to be performed.

The satisfactory outcomes of this case report agree with those reported by the literature evidencing that the sealed dentin is capable of remineralizing due to changes in the microenvironment [2] caused by the lack of substrates for the bacteria [3, 30]. Thus, new strategies for the management of the carious tissue may target alternative approaches in the treatment of the most advanced stages of the carious lesion, especially with benefits for the children.
